# Modified Littler Flap Reconstruction of the Small Finger

**Published:** 2020-01-09

**Authors:** Rebecca C. O'Neill, Ronald N. Bogdasarian, Ramazi O. Datiashvili

**Affiliations:** Division of Plastic and Reconstructive Surgery, Department of Surgery, Rutgers New Jersey Medical School, Newark

**Keywords:** Littler flap, small finger defects, thumb pulp defects, sensation restoration, hand reconstruction

**Figure:**
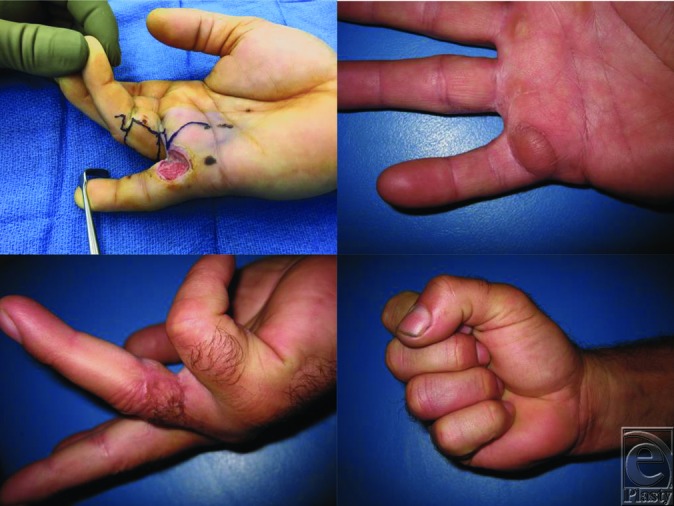


## DESCRIPTION

A 43-year-old, right-hand-dominant male truck driver presented after a low-voltage electrical burn to the right hand, resulting in a 2 × 2-cm open wound at the volar aspect of the proximal phalanx of the small finger with exposed tendon.

## QUESTIONS

1. What is the Littler flap?
2. What are the outcomes of Littler flap reconstruction?
3. What are the disadvantages of the Littler flap?
4. What modifications have been made over time to this technique?

## DISCUSSION

The hand's dexterity and sensory capacity endow humans with a powerful tool to manipulate the environment. There are a myriad of techniques to reconstruct open wounds of the hand. The Littler flap is a neurovascular island flap, originally described to reconstruct the open wound and restore sensation of the pulp following traumatic injury to the thumb.^1^ The donor site for the flap is typically the ulnar aspect of the middle or the radial aspect of the ring finger.^2^ The flap technique, as first described by Dr J. William Littler, utilizes the proper digital artery and proper digital nerve as the neurovascular pedicle of the island flap. The flap is raised from distal to proximal until the bifurcation of the common digital artery is reached.^1^^,^^3^ The flap is then passed under a subcutaneous tunnel to the recipient wound. The donor site is closed with a full-thickness skin graft.

The outcomes of the Littler flap are favorable. They are robust, reliable flaps, with high rates of survival. In most cases, the range of motion and cosmetic appearance of the thumb are deemed good to excellent.^3^^-^5 In these studies, cosmesis was evaluated by the surgeon subjectively or by patient satisfaction surveys and the range of motion was measured in comparison with the noninjured hand.^3^^,^^5^ The Littler flap enables patients to maintain 2-point discrimination sense, with mild reductions in light touch, evaluated by the Semmes-Weinstein monofilament test.^3^^-^5 Notably, no studies have used standardized scores to evaluate the functional results of the Littler flap.

The Littler flap is a suitable choice for larger defects of the volar thumb with concomitant nerve injury, as it is a single-staged procedure that provides a vascularized, sensate, pliable skin with a good cosmetic match for the thumb. Disadvantages of the Littler flap include the time required for cortical reorganization of sensation and donor site morbidity. A majority of the patients experience cold intolerance as well as reduced arterial flow to the donor digit in the postoperative period.^3^^,^^5^ Donor digit stiffness caused by immobilization has been significantly reduced with early mobilization.^4^ In addition, the Littler flap is technically challenging and requires a relatively steep learning curve.^4^

Several modifications of the Littler flap have been described. These include the transection and microsurgical coaptation of the nerve of the flap to the recipient digital nerve. This modification was performed to avoid the cortical reorganization. One study has demonstrated improved 2-point discrimination with this modification compared with the traditional technique.^5^

In our case, we modified the Littler flap for coverage of a small finger proximal segment wound with exposed flexor tendon. A 2 × 2-cm flap based on of the ulnar aspect of the proximal phalanx of the ring finger proximal segment was designed. The neurovascular bundle was identified distally, the flap was raised, and its pedicle was dissected to the axis of rotation near the takeoff point of the proper digital artery. The flap was transposed to the small finger and inset. The patient has done well postoperatively, with excellent excursion of the small finger flexor tendons and preserved sensation. Our case demonstrates that a modified Littler flap can provide robust coverage of adjacent finger and hand wounds.

In summary, the Littler flap is a neurovascularized flap designed to restore sensation to the thumb pulp but can be modified for most finger pulp wounds. Outcomes of the Littler flap are favorable, with improved range of motion with early rehabilitation and good cosmetic appearance. In our case, the advantages were durable coverage of exposed flexor tendon and provision of sensation to the small finger.
